# Mechanosensitive Pannexin 1 Activity Is Modulated by Stomatin in Human Red Blood Cells

**DOI:** 10.3390/ijms23169401

**Published:** 2022-08-20

**Authors:** Sarah Rougé, Sandrine Genetet, Maria Florencia Leal Denis, Michael Dussiot, Pablo Julio Schwarzbaum, Mariano Anibal Ostuni, Isabelle Mouro-Chanteloup

**Affiliations:** 1 Université Paris Cité and Université des Antilles, INSERM U1134, BIGR, F-75014 Paris, France; 2 Instituto de Química y Fisico-Química Biológicas “Prof. Alejandro C. Paladini”, UBA, CONICET, Facultad de Farmacia y Bioquímica, 1113 Buenos Aires, Argentina; 3 Université Paris Cité, INSERM U1163, IMAGINE, F-75015 Paris, France

**Keywords:** erythrocyte, ATP release, pannexin activity, stomatin, overhydrated hereditary stomatocytosis

## Abstract

Pannexin 1 (PANX1) was proposed to drive ATP release from red blood cells (RBCs) in response to stress conditions. Stomatin, a membrane protein regulating mechanosensitive channels, has been proposed to modulate PANX1 activity in non-erythroid cells. To determine whether stomatin modulates PANX1 activity in an erythroid context, we have (i) assessed the in situ stomatin-PANX1 interaction in RBCs, (ii) measured PANX1-stimulated activity in RBCs expressing stomatin or from OverHydrated Hereditary Stomatocytosis (OHSt) patients lacking stomatin, and in erythroid K562 cells invalidated for stomatin. Proximity Ligation Assay coupled with flow imaging shows 27.09% and 6.13% positive events in control and OHSt RBCs, respectively. The uptake of dyes 5(6)-Carboxyfluorescein (CF) and TO-PRO-3 was used to evaluate PANX1 activity. RBC permeability for CF is 34% and 11.8% in control and OHSt RBCs, respectively. PANX1 permeability for TO-PRO-3 is 35.72% and 18.42% in K562 stom^+^ and stom^−^ clones, respectively. These results suggest an interaction between PANX1 and stomatin in human RBCs and show a significant defect in PANX1 activity in the absence of stomatin. Based on these results, we propose that stomatin plays a major role in opening the PANX1 pore by being involved in a caspase-independent lifting of autoinhibition.

## 1. Introduction

Pannexin 1 (PANX1), a plasma membrane pore-forming channel, is widely expressed in various cell types and tissues [1]. In contrast to other members of the gap junction protein family, this single-membrane channel is no longer considered to form intercellular channels but rather described as exerting a physiological role mainly in nucleotide release [2]. In apoptotic cells, activated PANX1 has been proposed to mediate a ‘find-me’ signal for cell clearance by phagocyte recruitment [3]. In these cells, the most commonly accepted molecular mechanism of PANX1 activation is an irreversible cleavage by caspases 3 or 7 of its C-terminal (CT) tails [3,4,5], leading to a lift of autoinhibition by releasing the CT tails from the pore of the previously physically plugged channel.

The heptameric structure of PANX1 has been recently solved [6,7,8,9,10,11], giving new insights into the channel conformation though none of the presented structures could provide a precise visualization of the CT tail. Moreover, the CT tail cleavage cannot explain the PANX1 function under most physiological situations [12], where PANX1 is reversibly activated by several ligands in the absence of caspase activation. Such a mode of pore activation might entail a displacement of the CT tail due to sequential posttranslational modifications and/or binding of diverse stimuli on PANX1 subunits [12,13]. Alternatively, conformational changes in other parts of the macromolecule might participate in the gating mechanism. In this respect, dynamic conformational changes of the N-terminus of PANX1 have been associated with lipid movement in and out of the pore and with modifications of the functionality, suggesting a role in this domain of the gating mechanism of the membrane protein [14]. Among efficient stimuli tested in vitro, the most commonly described are pressure/stretch, hypotonicity, and increased extracellular K^+^ or intracellular Ca^2+^ [13]. PANX1 activation has been monitored by quantifying membrane currents, the release of intracellular ATP, and/or dye permeation [15,16,17]. However, K^+^ and Ca^2+^ have recently been shown to have no direct effect on PANX1 conformation [11].

Previous studies revealed the expression of PANX1 at the human and mouse erythrocyte membranes [18,19,20,21], and others showed that it is the main channel regulating ATP release from RBCs in response to either hypotonic and high K^+^ stress [17,22] or pharmacological and physiological stimuli [23,24]. In human, mouse, and Xenopus RBCs [25], ATP release directly correlated with the uptake of dyes with an exclusion limit >1 kDa. Moreover, RBCs from wild-type (WT) mice exhibited an activation of both ATP efflux and 5(6)-carboxyfluorescein (CF) uptake when stimulated with hypotonic high K^+^ medium, whereas RBCs from PANX1 deficient mice (Panx1^−/−^) did not take up the dye under similar conditions [21]. More recently, in response to hemoglobin deoxygenation (physiological stimulus), a significant lack of ATP export has been reported in vivo Panx1^−/−^ mice compared to WT [26].

PANX1 was suggested to react to local modifications or deformations of its membrane environment [15]. In the erythroid context, we wondered whether the integral membrane protein stomatin might directly interact with PANX1, affecting its function. A previous report showed an interaction between recombinant proteins PANX1 and stomatin co-expressed in HEK293 cells, highlighting the involvement of the CT tail of PANX1 in this interaction [27]. Moreover, stomatin is known to interact with various ion channels and modulate their activities [28]. As regards the acid-sensing ion channel 3 (ASIC3) in sensory neurons, an accurate relationship was described between the inhibition of the channel and the oligomeric state of the SPFH (stomatin, prohibitin, flotillin, HflC/K)-domain of stomatin [29,30], a domain then reported to be common to stomatin-like and related proteins [31]. Interestingly, the molecular mechanism of this inhibition has recently been highlighted [32]. Stomatin has also recently been shown to interact with the Na^+^ taurocholate cotransporting polypeptide (NTCP) in hepatocytes and to modulate bile salt uptake [33]. In RBCs, stomatin is particularly well expressed and localized in cholesterol-rich lipid rafts, with connections to actin and the spectrin-based skeleton [34]. The monomeric form of stomatin, also known as protein 7.2 or as the major component of band 7, exhibits an apparent molecular weight of 31-kDa [35,36] and is totally absent from mature RBCs of a rare human haemolytic anaemia, hereditary stomatocytosis (OHSt) [37]. Previous studies clearly identified the interaction between stomatin and several channels or transporters in erythrocyte membrane domains [38]. They demonstrated its capability to regulate the activity of membrane transporters in RBCs, such as the glucose transporter GLUT-1 and the anion exchanger Band 3 (or AE1) [39,40].

In this study, we characterized, for the first time, a physical and functional link between PANX1 and stomatin at the human RBC membrane. We used an in situ Proximity Ligation Assay (PLA) to highlight the colocalization (<40 nm) of stomatin and PANX1 at the RBC membrane. We also took advantage of samples from three OHSt patients previously described to naturally lack stomatin [41] to investigate the role of stomatin on PANX1 activity. Finally, we confirmed our results by measuring PANX1 activity on both WT K562 (stom^+^), and K562 invalidated for stomatin (stom^−^).

## 2. Results

### 2.1. Deformability Properties over an Osmotic Gradient Measured in RBCs from Controls and OHSt Patients

**Figure 1:**
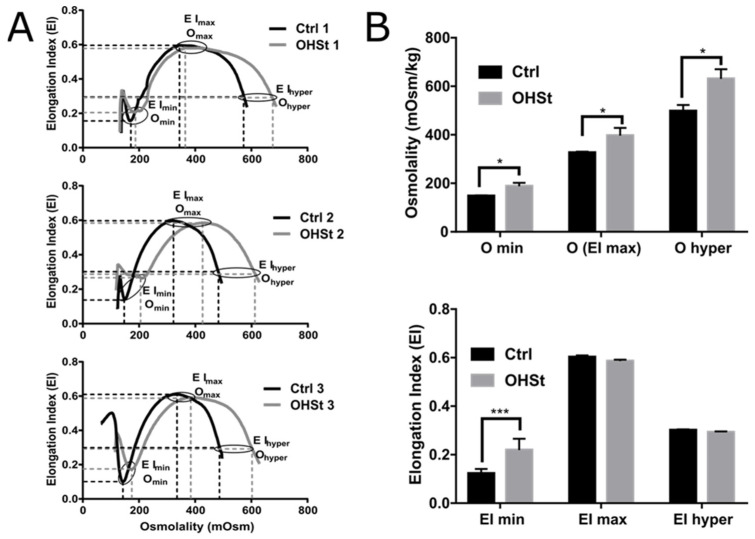
Rheological properties of OHSt RBCs compared to controls. (**A**) Osmoscan curves of the 3 studied OHSt patients (in grey) compared to healthy control (Ctrl) subjects (in black) with critical points (Osmolality: O_min_, O_max_, O_hyper_ and Elongation Index: EI_min_, EI_max_, EI_hyper_) specified on each curve. (**B**) The values of these critical points have been determined, and their averages have been compared between OHSt and Ctrl using a multiple t-test (*p* values ≤ 0.05: * and ≤0.001: ***).

Osmotic gradient ektacytometry (osmoscan) was used to determine RBC deformability or the Elongation Index (EI) under defined shear stress as a function of medium osmolality (in mOsm/kg). Osmoscan curves are similar for each OHSt patient compared to appropriate controls (Figure 1A). A characteristic shift to the right was observed with Osmolality values significantly higher for the patient RBCs (O_min_: 187.7 vs. 147.6 mOsm/kg, O(EI_max_): 396.3 vs. 326.0 mOsm/kg and O_hyper_: 630.3 vs. 497.3 mOsm/kg, for OHSt and Control, respectively) (Figure 1B), indicating an osmotic fragility and overhydration, as previously described in OHSt RBCs [42,43]. The Elongation Index values were similar among all the tested RBCs from both controls and OHSt patients except for EI_min_, which was significantly higher for OHSt (0.219 vs. 0.122 in controls) (Figure 1B). This result might be related to the O_min_ increase in the patient RBCs.

### 2.2. Stomatin and PANX1 Expression at the RBC Membrane of Controls and OHSt Patients

**Figure 2:**
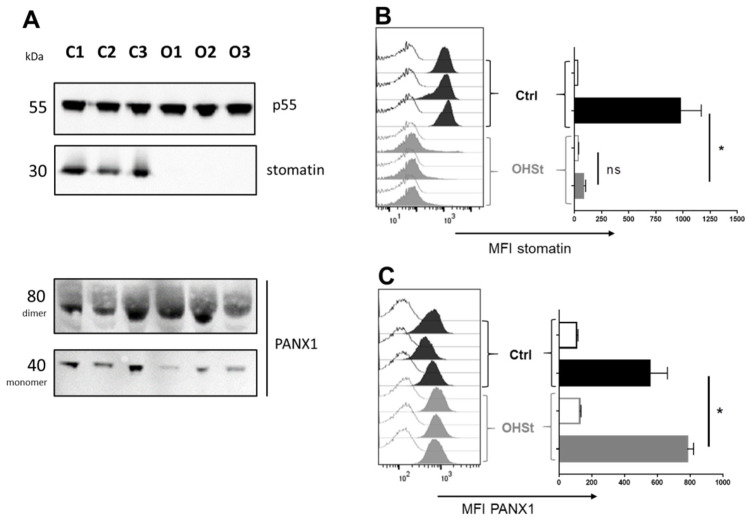
Stomatin and PANX1 expression in OHSt RBCs compared to controls. (**A**) Western blot analysis of RBC ghosts from 3 controls (C1, C2, C2) and the 3 OHSt patients (O1, O2, O3) using mouse monoclonal anti-stomatin (E-5), rabbit polyclonal anti-PANX1 and rabbit polyclonal anti-p55 as control. The molecular weights (kDa) of the detected bands are mentioned. (**B**) Flow cytometry analysis of stomatin expression in the RBCs of the 3 controls and the 3 OHSt patients after permeabilisation. Negative controls consist of the observed fluorescence with only the secondary antibody. The averages of Mean of Fluorescence Intensity (MFI) were compared between OHSt and control (Ctrl) RBCs using a two-way ANOVA Sidak’s multicomparison test (*p* values > 0.05: ns and ≤0.05: *, ns stands for not significant), paired for comparisons within OHSt samples when signals with anti-stomatin were compared to that of Negative controls and unpaired when OHSt were compared to Ctrl. (**C**) Flow cytometry analysis of PANX1 expression in the 3 controls and the 3 OHSt patient RBCs after permeabilization and comparisons of MFIs between controls and OHSt, as performed in B.

Western blot analysis confirmed the expected lack of stomatin in OHSt samples (Figure 2A), using a mouse monoclonal antibody (E-5, see material and methods) raised against amino acids 1–45 mapping at the intracellular N-terminus of the human stomatin. Similarly, using a KO-validated rabbit polyclonal antibody raised against amino acids 18–31 mapping at the intracellular N-terminus of PANX1 revealed the presence of PANX1 at the RBC membrane of control and OHSt patients (Figure 2A). On the immunoblot, PANX1 could be detected at the expected molecular weight of 40 kDa representing a monomeric form. Moreover, large bands at 80 kDa were also detected in all samples, and these bands most probably correspond to a dimer of PANX1. We used the same antibodies in flow cytometry analysis to measure stomatin and PANX1 expression levels in permeabilized RBCs from controls and OHSt patients. The values of Mean Fluorescence Intensity (MFI) confirmed the stomatin-deficiency in OHSt RBCs (MFI: 85.8 vs. 978.3 in Controls). Regarding the expression level of PANX1, this study revealed a slight increase in OHSt (MFI: 783) compared to controls (MFI: 553) (Figure 2B), suggesting either a higher PANX1 copy number at the OHSt RBC membrane or increased accessibility of the anti-PANX1 antibody in the absence of stomatin. A similar trend was found when quantifying the expression level of AE1 on OHSt RBC membranes with anti-AE1 (unpublished results). Even though the specificity of the anti-PANX1 antibody signal has been previously demonstrated by the supplier using KO samples, it was also validated here by flow cytometry using RBCs previously incubated with a competitor peptide (Figure S1).

### 2.3. Proximity between Stomatin and PANX1 in Control RBCs

**Figure 3:**
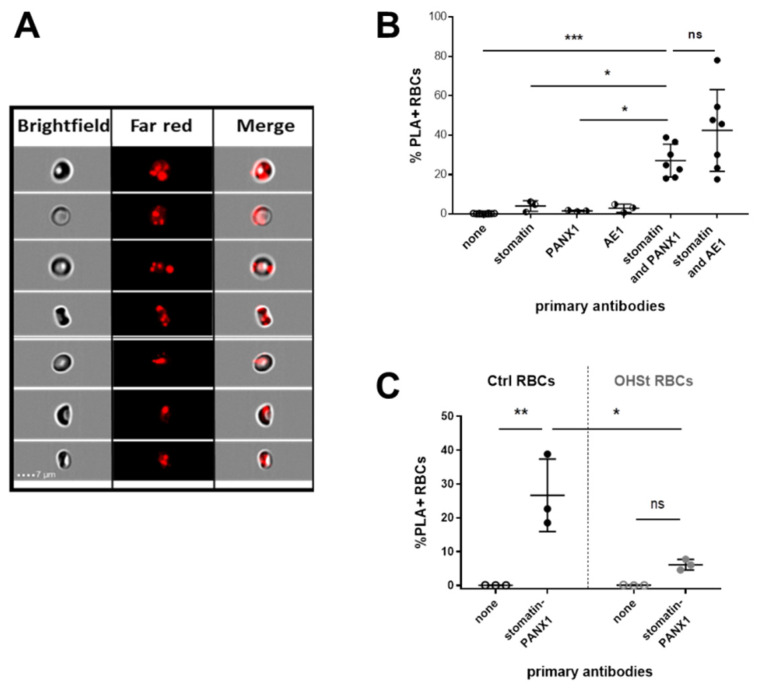
Stomatin and PANX1 proximity in control and OHSt RBCs assessed by Proximity Ligation Assay (PLA) and analyzed by Imaging Flow Cytometry. (**A**) PLA-positive (PLA+) events characterized by red spots which result from the detection oligos coupled to fluorochromes (FarRed) hybridizing to sequences in the amplified DNA were visualized by Imaging Flow Cytometry and merged with Brightfield images of the corresponding Control RBCs. (**B**) Quantification of the assay corresponding to the percentage of PLA+ RBCs was performed several times in different conditions: without any primary antibody (none, N = 10), with only one antibody (anti-stomatin or anti-PANX1 or anti-AE1, N = 3 for each condition) and in the presence of two antibodies (anti-stomatin + anti-PANX1 or anti-stomatin + anti-AE1, N = 7 in both conditions). A comparison was performed using an unpaired One-way Anova Tukey’s test. (**C**) PLA using anti-stomatin + anti-PANX1 was performed in identical conditions to those described above but on frozen/thawed control (Ctrl) and OHSt RBCs and compared using paired or unpaired (between control and OHSt) Two-way Anova Sidak’s multiple comparisons test (*p* values > 0.05: ns, ≤0.05: *, ≤0.01: ** and ≤0.001: ***). ns stands for not significant.

Using validated anti-stomatin and anti-PANX1 antibodies, an in situ PLA was performed on fixed and permeabilized control red cells from unfrozen control blood of 7 different individuals. Following the incubation with the primary antibodies, the addition of PLA reagents and successive steps (probe coupling, ligation, polymerization, and finally hybridization with a fluorescent dye) produced positive reactions that were characterized by FarRed spots clearly visualized in some RBCs using an Amnis ImageStream Imaging Flow Cytometer (Figure 3A). The positive reactions corresponding to the proximity of 40 nm or less between the two stomatin and PANX1 proteins could be quantified by determining a percentage of RBCs containing the FarRed spots designated as %PLA+. The anti-stomatin/anti PANX1 antibody pair gave 27.09% PLA+ (Figure 3B). This result could be compared to that obtained in the same conditions but using the anti-stomatin/anti-AE1 antibodies, which gave 42.38% PLA+ (Figure 3B), in agreement with previous studies [39]. The slightly higher percentage obtained for the positive control is consistent with the high expression level of both stomatin and AE1 compared to a much lower expression level of PANX1 in RBCs. In this assay, negative controls were initially composed of reactions missing one of the two primary antibodies or both, giving 4.01% PLA+ and 1.57% PLA+, when anti-stomatin and anti-PANX1 were used alone, respectively (Figure 3B). Moreover, we used stomatin-deficient RBCs to perform another PLA negative control, this time using the anti-stomatin/anti-PANX1 antibody pair. This PLA was performed on frozen and thawed RBCs and resulted in 6.13% PLA+ from the RBCs of the 3 OHSt patients (Figure 3C). This value was significantly lower than that obtained from frozen and thawed control RBCs (26.67%). Interestingly, values for the stomatin/PANX1 tests were very similar whether control RBCs had been frozen/thawed or not (27.09% vs. 26.67% PLA+, respectively).

### 2.4. ATP Release from RBCs

Rates of intracellular ATP release from RBCs were estimated by quantifying eATP concentration by online luminometry. The first series of experiments were run on fresh RBCs.

**Figure 4:**
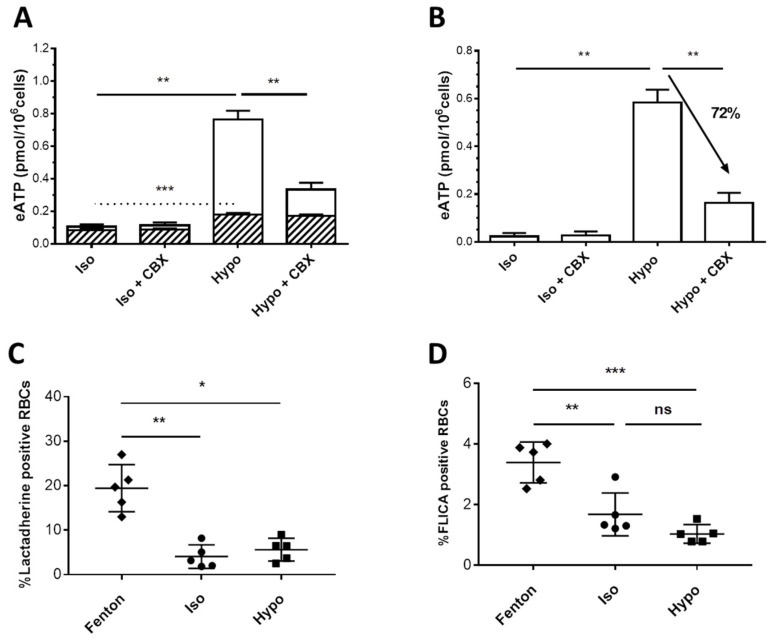
Activation of ATP release in fresh RBCs by a hypoosmotic challenge. (**A**) RBC suspensions were exposed to isoosmotic (Iso) or hypoosmotic (Hypo, 240 mOsm/kg) stimulation. Experiments were performed with or without preincubation with 100 μM CBX, a PANX1 inhibitor. Results are expressed as eATP (pmol/10^6^ cells). The hemoglobin concentration of paired samples was used to estimate lytic eATP. A stacked graph was built to show the contribution of lytic ATP (hatched bars) within total ATP release from RBCs. Lytic and total eATP values were compared using a One-Way ANOVA Dunnett’s test for Multiple Comparisons (N = 5). The results shown are mean values ± SEM. (**B**) The difference between total eATP and lytic eATP was calculated and denoted as non-lytic eATP. Non-lytic eATP values were compared using a One-Way ANOVA Dunnett’s test for Multiple Comparisons. (**C**) PS exposure was measured by flow cytometry on Lactadherin-FITC stained RBCs in basal conditions after treatment with Fenton’s reagent, as a control of eryptosis, and in basal or hypoosmotic conditions without Fenton. (**D**) Caspase activation was measured under conditions similar to PS exposure but on FLICA 660 caspase3/7-stained RBCs. The statistical analysis of the last two measurements (N = 5) was performed using an RM-one-way ANOVA test. (*p* values > 0.05: ns, ≤0.05: *, ≤0.01: ** and ≤0.001: ***). ns stands for not significant.

In isoosmotic medium (iso), basal levels of eATP in RBCs amounted to 0.10 ± 0.02 pmol/10^6^ cells (Figure 4A). The hypoosmotic treatment (hypo) led to a 7-fold increase in total eATP concentration. In contrast, after stimulation, the increase of lytic ATP accounted for only 13% of the hypotonically induced-ATP release. Carbenoxolone (CBX), a well-known PANX1 inhibitor, significantly reduced the hypotonically elevated total eATP content by 56% (i.e., from 0.76 to 0.33 pmol/10^6^ cells) (Figure 4A).

These results suggest that in fresh RBCs, most ATP release is mediated by PANX1. Fresh RBCs showed neither PS externalization nor caspase activation (Figure 4C,D), thus suggesting that PANX1 activation by hypotonicity does not require an irreversible caspase-dependent CT tail cleavage.

ATP release was also assessed in frozen/thawed RBCs from OHSt RBCs and their respective frozen/thawed control RBCs. Prior to experiments, cells were thawed and rejuvenated to increase levels of intracellular ATP and 2,3-diphosphoglycerate (2,3-DPG) to that of fresh cells [44].

**Figure 5:**
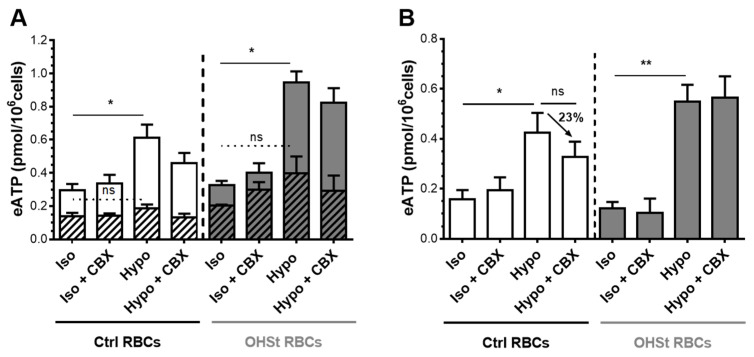
Activation of ATP release in frozen/thawed and rejuvenated RBCs from controls and OHSt patients. Suspensions of frozen/thawed and rejuvenated RBCs were exposed to isoosmotic (Iso) or hypoosmotic (Hypo, 240 mOsm/kg) stimulation. Experiments were performed with or without preincubation with 100 μM CBX, a PANX1 inhibitor. Results are expressed as eATP (pmol/10^6^ cells). (**A**) Stacked graph showing total eATP including lytic eATP (hatched bars) released from control (white bars) or OHSt (grey bars) RBCs. The hemoglobin concentration of paired samples was used to estimate lytic eATP. (**B**) Non-lytic eATP was calculated as the differences between total eATP and lytic eATP. Total, lytic, and non-lytic eATP values were compared using a One-Way ANOVA Dunnett’s test for Multiple Comparison (N = 3). The results shown are mean values ± SEM. (*p* values > 0.05: ns, ≤0.05: *, ≤0.01: **). ns stands for not significant.

The hypoosmotic treatment triggered a 2- and 2.9-fold increase of eATP in control and OHSt RBCs, respectively, suggesting activation of ATP release (Figure 5A). Baseline (Iso) eATP was equivalent between control and OHSt RBCs. However, these values are significantly higher than baseline values obtained using fresh RBCs (Figure 4B). Furthermore, exposure to 100 µM CBX did not significantly affect the hypoosmotically stimulated ATP release in both cell groups and was clearly less efficient than in fresh RBCs (decrease of 23%, Figure 5B control cells, compared to 72%, Figure 4B). Results suggest that in frozen/thawed rejuvenated RBCs, PANX1 is not the main conduit mediating ATP release in response to hypoosmotic stimulation.

### 2.5. 5(6)-Carboxyfluorescein (CF) Uptake after High K^+^ Stimulation of Control and OHSt RBC Membranes

Because frozen/thawed cells had lost the ability to respond to hypoosmotic stimulation, we decided to stimulate these cells using a high K^+^ buffer as previously described [17]. Uptake of the fluorescent tracer molecule 5(6)-carboxyfluorescein by human erythrocytes was performed under conditions where Na+ was replaced by K^+^ without changing the osmolarity of the Krebs solution. The Mean Fluorescence Intensity (MFI) was determined by flow cytometry following the protocol described in the patients and methods section below.

**Figure 6:**
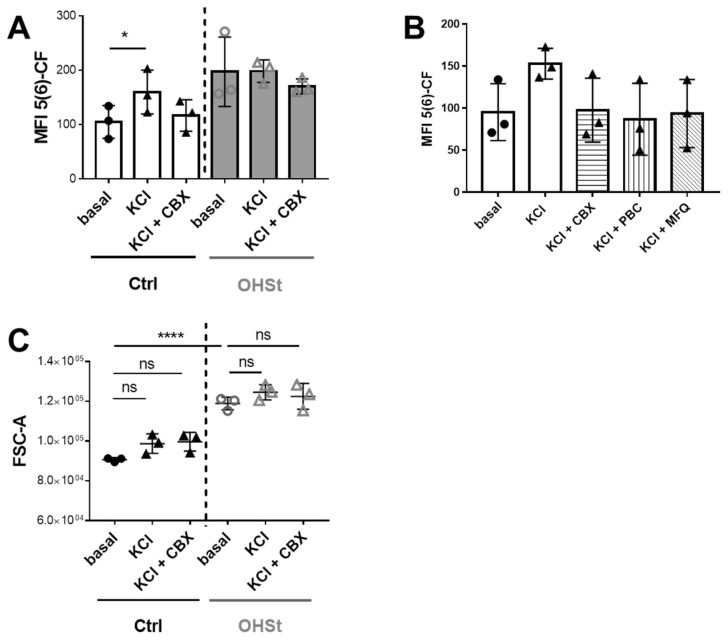
5(6)-carboxyfluorescein uptake in control (Ctrl) and OHSt RBCs after stimulation by high K^+^. (**A**) MFI due to the dye uptake in Ctrl (black) and OHSt (grey) frozen/thawed rejuvenated RBCs measured by flow cytometry in basal conditions and after stimulation by KCl without or with incubation with CBX. (**B**) Carbenoxolone (CBX), probenecid (PBC), and mefloquine (MFQ), three different PANX1 inhibitors, were added to RBC 20 min before the stimulation, resulting in a fluorescence signal similar to that of basal conditions. (**C**) FSC was also determined in all the conditions described above under KCl stimulation. Data obtained from stimulated cells were compared to that of unstimulated cells or cells stimulated in the presence of CBX using a paired Two-way Anova Sidak’s multiple comparisons test. The same test but unpaired was used when Ctrl and OHSt RBCs were compared. (*p* values > 0.05: ns, ≤0.05: *, ≤0.0001: ****). ns stands for not significant.

For frozen/thawed control RBCs, a significant 34% increase in fluorescence intensity was observed in the presence of K^+^ (MFI Basal: 105 vs. MFI KCl: 160) (Figure 6A, white bars), and the use of three different PANX1 inhibitors resulted in a fluorescence signal was similar to that of basal conditions (Figure 6B). However, the RBC volume, corresponding to the FSC intensity (that is proportional to the diameter of the cells), was unmodified after stimulation (Figure 6C, black symbols). These results strongly indicated that PANX1 is activated in control RBCs by KCl. In contrast, on frozen/thawed OHSt RBCs, after KCl stimulation, the increase of fluorescence intensity was very weak and not significant (Figure 6A, grey bars). Moreover, the decrease of fluorescence intensity after preincubation with CBX was also non-significant. When frozen/thawed OHSt RBCs were compared to control RBCs in terms of their size, an expected significant increase of 31% (FSC basal OHSt: 118915 vs. FSC basal control: 90647) (Figure 6C, white symbols) was observed, consistent with the well-known increased volume of OHSt RBCs compared to that of control RBCs (in this study, mean RBC volume for OHSt: 138.2fl and for controls: 90fl, Supplementary Table S1). In high K^+^ conditions, the size of OHSt RBCs was also unmodified. All these results strongly indicate that stomatin-deficient OHSt RBCs appear insensitive to K^+^ stimulation regarding the fluorescein uptake, suggesting a potential inactivation of PANX1 in these cells.

### 2.6. Endogenous Expression of Stomatin and PANX1 in K562 Cells and PANX1-Dependent TO-PRO-3 Uptake

As we could not completely exclude that OHSt RBCs display other membrane abnormalities besides stomatin deficiency, we decided to test PANX1 activity using the nucleated erythroid K562 cell line. As we show below, using this cell line, it was possible to knock down the *STOM* gene and compare PANX1 activity in WT (stom^+^) vs. stom^−^ cells.

**Figure 7:**
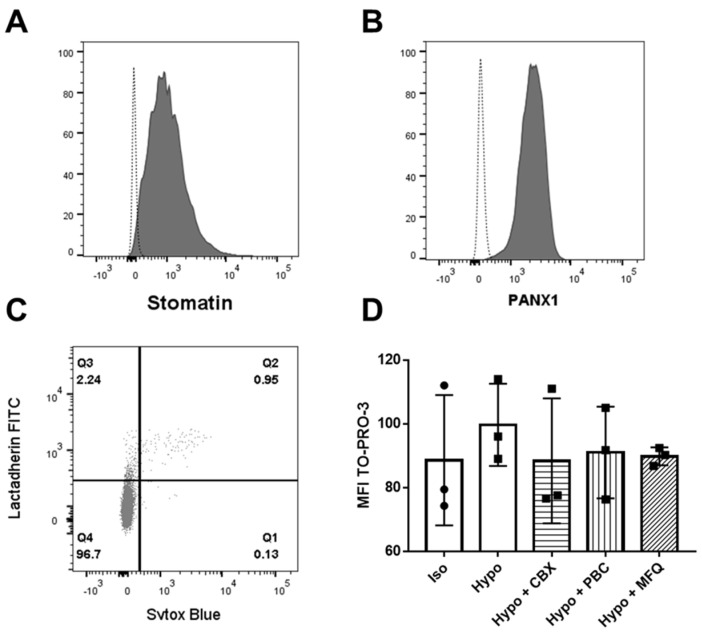
Endogenous expression of stomatin and PANX1 in K562 cells and PANX1-dependent TO-PRO-3 uptake by non-apoptotic K562 cells. (**A**) Stomatin expression analysed by flow cytometry on permeabilized K562 cells using anti-stomatin E5 (Santa Cruz Biotechnology, Dallas, TX, USA). (**B**) PANX1 expression analysed by flow cytometry on permeabilized K562 cells using anti-PANX1 (Alomone Labs, Jerusalem, Israel). (**C**) Gating strategy of Sytox-negative (Pacific Blue) and Lactadherin-negative (FITC) K562 cells (at least 85%, Q4) in which the TO-PRO-3 staining (APC) was evaluated by flow cytometry. (**D**) TO-PRO-3 uptake was measured on K562 cells in basal conditions and, after a hypotonic stimulation (80 mOsm/kg), on K562 cells that were preincubated or not with several PANX1 inhibitors (carbenoxolone [CBX], probenecid [PBC], and mefloquine [MFQ]) (N = 3).

Endogenous expressions of stomatin and PANX1 in K562 cells were tested by flow cytometry with the anti-stomatin and anti-PANX1 antibodies previously used on RBCs. Clear positive signals (MFI anti-stomatin: 1013 and MFI anti-PANX1: 2153) were obtained (Figure 7A and Figure 7B, respectively) compared to controls.

In these cells, PANX1 activity was assessed by the uptake of the FarRed fluorescent tracer molecule TO-PRO-3, as previously described [4], after stimulation by hypotonic stress of 80 mOsm. However, TO-PRO-3, impermeant to live cells, can penetrate cells not only through activated PANX1 channels but also through compromised membranes characteristic of dead cells. Importantly, these dead cells had to be excluded from the analysis of TO-PRO-3 uptake, and this could be done by detecting their specific positive stain with Sytox blue. In addition, the apoptotic cells exhibiting a possible irreversible activation of PANX1 by caspase cleavage of the CT tail also had to be excluded. They were specifically stained with Lactadherin FITC, detecting extracellular phosphatidylserine. The TO-PRO-3 uptake was analyzed on at least 85% of viable K562 clones (Sytox-negative and Lactadherin-negative, Figure 7C).

Preincubation of K562 cells with several PANX1 inhibitors (carbenoxolone [CBX], probenecid [PBC], and mefloquine [MFQ]) before hypotonic stimulation resulted in fluorescence intensities comparable to that obtained in isotonic conditions (Figure 7D CBX: 88.37, PBC: 91, MFQ: 89.8 compared to control Iso: 88.57). This indicates that the increase of TO-PRO-3 fluorescence after stimulation is PANX1 dependent.

### 2.7. Generation of Stomatin CRISPR/Cas9 Knockout K562 Cell Clones

**Figure 8:**
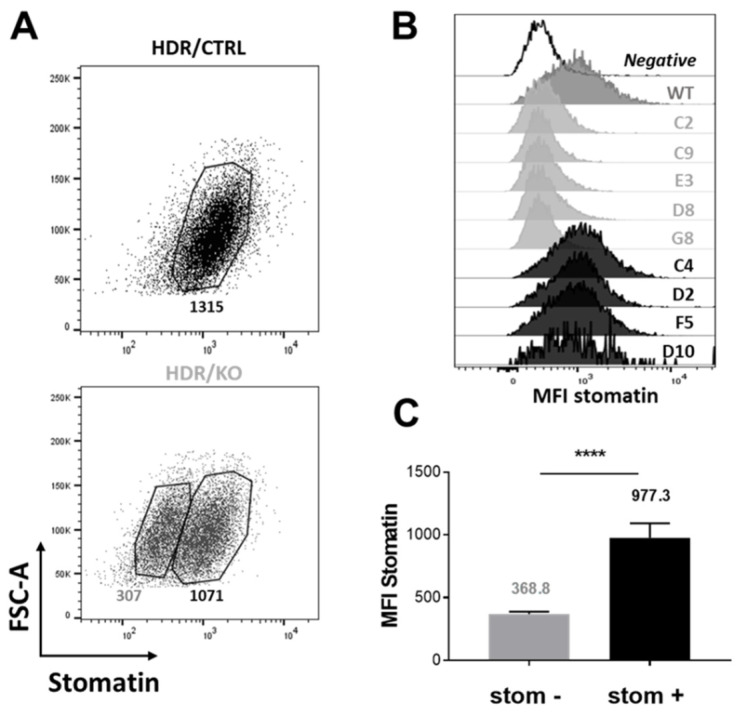
Stomatin deficiency on stomatin Knockout transfected K562 cells and generation of stom^+^ and stom^−^ clones. (**A**) Stomatin expression was assessed by flow cytometry on puromycin-resistant K562 cells after co-transfection with stomatin Homology-Directed DNA Repair (HDR) plasmids together with either control CRISPR/Cas9 (CRISPR Control) or stomatin CRISPR/Cas9 Knockout (CRISPR KO) plasmids. This led to two different dot plot representations (HDR/ CRISPR Control and HDR/ CRISPR KO), where fluorescence intensity corresponding to stomatin expression was plotted on the x-axis and Forward Scatter (FSC) on the y-axis. (**B**) Flow cytometry profiles of K562 stomatin-negative clones (C2, C9, E3 D8, and G8) compared to stomatin-positive controls (WT) and controls incubated without the primary anti-stomatin antibody (negative). (**C**) MFI averages of stomatin-negative (stom^−^) and stomatin-positive (stom^+^) K562 clones. A comparison of their stomatin expression level was performed using an unpaired *t*-test. (*p* values ≤ 0.0001: ****).

After co-transfecting K562 cells with stomatin Homology-Directed DNA Repair (HDR) plasmids with either control CRISPR/Cas9 or stomatin CRISPR/Cas9 Knockout plasmids, cells were stabilized for 3 weeks in the presence of puromycin for selection of cells where Cas9-induced DNA cleavage had occurred. The puromycin-resistant cells from both co-transfections were tested by flow cytometry after permeabilization and incubation with an anti-stomatin antibody (Figure 8A). Cells co-transfected with HDR and control CRISPR/Cas9 plasmids were distributed into a homogenous population expressing stomatin (MFI: 1315). In contrast, cells co-transfected with HDR and stomatin CRISPR/Cas9 KO plasmids were distributed into two sub-populations, one showing a similar expression level of stomatin to the control cells (MFI: 1071) and the second one exhibiting a marked decrease in stomatin expression (MFI: 307). A limiting dilution of these cells gave rise to several clones. A flow cytometry analysis of each clone regarding stomatin expression clearly showed that each was derived from one of the two sub-populations (Figure 8B). Five clones (C2, C9, E3 D8, and G8) were identified as stomatin-negative (stom^−^) with a stomatin expression level similar to that of the negative control (average MFI for negative control: 305 (not shown) and the average of MFI for the 5 clones: 368.8, Figure 8C).

### 2.8. PANX1 Expression and Caspase-Independent TO-PRO-3 Uptake in stom^+^ and stom^−^ K562 Cell Clones

**Figure 9:**
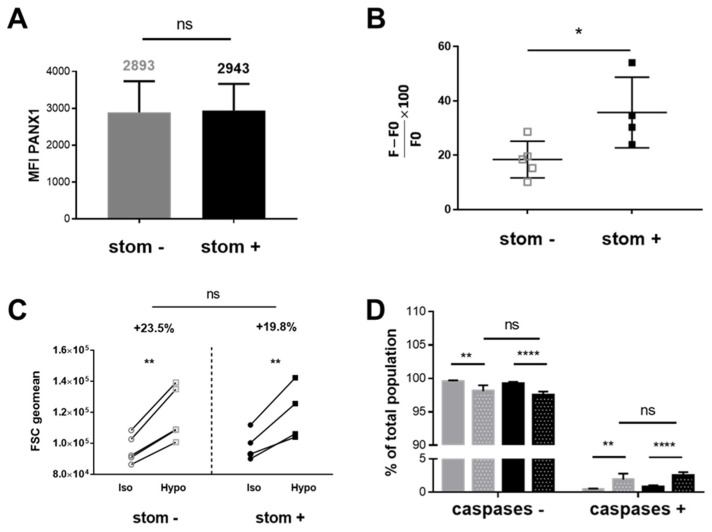
TO-PRO-3 uptake in viable and non-apoptotic stom^+^ and stom^−^ K562 cells after hypotonic stress. (**A**) Expression of PANX1 in stom^−^ and stom^+^ K562 clones was assessed by flow cytometry, giving similar levels. (**B**) Comparison of the relative TO-PRO-3 fluorescence intensity (F − F0/F0 − 100) between stom^+^ (black squares) and stom^−^ (grey squares) K562 cell clones using an unpaired t-test. (**C**) Cell volume of stom^+^ (black) and stom^−^ (grey) K562 clones assessed by flow cytometry comparing FSC geomean values obtained in isotonic (circles) and hypotonic (80 mosm/kg, squares) conditions. (**D**) Caspase 3/7 activity measured by flow cytometry analysis allowing the determination of the percentage of fluorescent stom^+^ (iso: black and hypo: black with white dots) and stom^−^ (iso: grey and hypo: grey with white dots) K562 cell clones preloaded by the NucView substrate and stimulated by hypotonic stress (two-way ANOVA Sidak’s multiple comparisons test). (*p* values > 0.05: ns, ≤0.05: *, ≤0.01: ** and ≤0.0001: ****).

A similar endogenous expression level of PANX1 was detected for the stom^+^ (average MFI: 2943) and the stom^−^ (averaged MFI: 2893) K562 clones (Figure 9A) compared to WT K562 cells (Figure 7B).

Under hypotonic stimulation, the average (three different measurements for each clone) of the fluorescence intensities due to TO-PRO-3 uptake was determined. In stomatin-negative clones, a significant decrease in fluorescence, as compared to stomatin-positive clones (normalized fluorescence (F − F0)/F0 × 100: 18.42 for stom^−^ and 35.72 for stom^+^) was observed, indicating that PANX1 is inactive in these clones (Figure 9B). Interestingly, the cell volume of stimulated K562 cells increased similarly whether they expressed stomatin (Figure 9C), ensuring accurate comparisons between the TO-PRO-3 fluorescence intensities measured in several conditions. As shown in Figure 9D, the same hypotonic stimulation led to a slight but significant activation of caspases 3/7 in both stom^+^ and stom^−^ K562 clones, respectively 2.49% and 1.90%, but this was not enhanced in the absence of stomatin.

## 3. Discussion

To study the functional properties of the mechanosensitive channel PANX1 and to explore its modulation by stomatin, we carried out investigations using RBCs and K562 cells. While stomatin is ubiquitously expressed at the membrane of both cell types, the cell surface expression of PANX1 has not been clearly identified to date. Indeed, in human RBCs, the presence of PANX1 has been essentially demonstrated by functional studies [17,24] or by western blot [18], and, in K562 cells, its expression has previously been reported from proteomic studies [45]. In the present study, we used a specific anti-PANX1 antibody which allowed a clear identification of PANX1 at the membrane of human RBCs and K562 cells. On RBCs, a glycosylated form of the channel was revealed, consistent with a glycosylation site previously described at the second extracellular loop (Asn-254, [46]). Glycosylation in PANX1 is considered essential to prevent two oligomers from docking and forming an intercellular channel, thus ensuring that they form a single-membrane channel connecting the intra- and extracellular compartments of cells.

Furthermore, to highlight proximity (<40 nm) between PANX1 and stomatin at the erythrocyte membrane, an in situ PLA coupled with imaging flow cytometry was used to visualize and quantify, for the first time in RBCs, PLA-positive events. The in situ stomatin-PANX1 proximity in RBCs revealed in this study is consistent with a previously reported interaction between the two recombinant proteins stomatin and PANX1 by an in vitro pull-down assay [27]. Since stomatin was reported as a major protein in cholesterol-rich microdomains of RBCs (lipid-rafts) [47], present data strongly suggest that PANX1-stomatin interactions occur in these microdomains of the RBC membranes.

Many stimuli have been described to activate PANX1 in a large number of cells expressing the endogenous or the recombinant mechanosensitive channel [13]. In the present study, when fresh RBCs were stimulated in vitro by applying hypotonic stress, a substantial non-lytic ATP release, sensitive to PANX1 inhibitor carbonoxolone (CBX), strongly indicated that PANX1 plays an essential role in ATP release from RBCs, as previously described with other stimuli [4,22,23,48]. However, a considerable release of ATP was also observed in unstimulated frozen/thawed and rejuvenated RBCs, not due to hemolysis. Furthermore, the stimulation by the hypotonic stress induced a small but significant increase of ATP release that could not be inhibited by CBX, suggesting other exit pathways than PANX1 for ATP. The voltage-dependent anion channel (VDAC) previously proposed to be able to release ATP once oligomerized [49] could be involved in this pathway, and the oligomerization state of VDAC would be interesting to investigate in frozen/thawed RBCs. The present study shows that in vitro ATP release assays, which can be successfully used to specifically study the PANX1 activity on fresh RBCs, are unsuitable for such investigations on frozen/thawed and rejuvenated RBCs. In contrast, stimulation by K^+^ previously described to induce 5(6)-Carboxyfluorescein dye uptake through the activated PANX1 [20] could be efficiently reproduced in this study on fresh as well as on frozen/thawed RBCs.

We took advantage of the availability of blood samples of 3 OHSt patients, genetically defined by a mutation in the *RHAG* gene [41] and phenotypically characterized by a cation leak and a stomatin deficiency [50,51,52]. In the absence of mutation in the *STOM* gene, the origin of this deficiency in mature erythrocytes is still unknown [53]. However, the rheological properties of the patient RBCs, as previously described for OHSt [43], resulted in specific values on the osmoscan curves that were characteristic of an increase in osmotic fragility and overhydration [54], and this could be compared for the first time between various OHSt patients. The present study also revealed a common EI_min_ value for the 3 OHSt, indicating an altered surface/volume of their RBCs. Importantly, the stomatin deficiency reasonably suggests that the activity of RBC membrane channels or transporters that are shown to interact with stomatin in control RBCs [38] could be impacted in OHSt, as previously described for GLUT1 [40] and AE1 [39]. Using frozen/thawed control and OHSt RBCs tested simultaneously, we have demonstrated that cells expressing both PANX1 and stomatin can uptake 5(6)-Carboxyfluorescein and that this uptake is inhibited by three well-characterised inhibitors of PANX1 belonging to different chemical families [55,56,57]. Moreover, OHSt lacking stomatin was not able to take up the dye. Altogether, the data presented in this study demonstrate that PANX1 is endogenously expressed in RBCs and colocalizes with stomatin which regulates PANX1 activity.

To confirm results obtained with stom^+^ (control) and stom^−^ (OHSt) RBCs, we used CRISPR Cas9 to generate K562 stom^−^ clones exhibiting unchanged endogenous PANX1 expression levels and no modification in their cell volume. In stom^−^ K562 clones, TO-PRO-3 uptake was measured by flow cytometry after a hypoosmostic (80 mOsm/kg) stimulation of cells. Uptake of this impermeant dye was previously associated with PANX1 activation in HEK293 cells [4]. However, Chui et al. described that the apoptotic process in these cells could be associated with the activation of caspases 3/7, which can cleave the CT tail end of PANX1 and, therefore, irreversibly open the channel [3]. Together with increased permeability to TO-PRO-3 in dying cells [58], this caspase activation can lead to a dye uptake independently of the mechanical stimulation. Unlike these previous studies focusing on the irreversible activation of PANX1, we were interested in investigating the role of stomatin in the caspase-independent activation of PANX1. The apoptotic and dead cells were therefore excluded from the TO-PRO-3 uptake analysis. When only viable cells were tested, the dye uptake was compared between stom^+^ and stom^−^ K562 clones, showing a significant decrease in fluorescence in the absence of stomatin. As for CF uptake in RBCs, the TO-PRO-3 uptake in K562 cells was strongly inhibited by 3 different PANX1 inhibitors, clearly indicating that this uptake is due to PANX1 activity. Interestingly, the hypoosmotic stimulation weakly but significantly induced the activation of caspases. However, caspase activation was similar in stom^−^ clones and could therefore not account for a lower dye uptake in these cells. Consequently, the stom^−^ K562 cells appeared less sensitive to the hypotonic stress regarding caspase-independent PANX1 activation.

All these results clearly demonstrate a role of stomatin, shown here to be in the proximity of PANX1, in this alternative small conductance ion-conducting pathway that involves side tunnels [11]. In addition, the recent description of a dynamic conformational change of the N-terminal ends in association with lipid movement *in* and *out* of the pore leading to modifications of the functionality [14] also supports the involvement of the stomatin. Indeed, this major lipid-raft protein at the RBC membrane could play an indirect role in this gating mechanism, probably by maintaining a specific lipid environment around the PANX1 membrane protein. In the future, it will be interesting to carry out ATP release studies on fresh stomatin-deficient RBCs from OHSt patients when they are available, as well as biochemical and structural characterization of native PANX1-stomatin complexes using recently developed detergent-free protocols specially adapted to studying RBC membrane proteins [59].

## 4. Methods and Materials

### 4.1. Control and Patient RBCs

Blood from healthy volunteers and 3 OHSt patients with the Phe65Ser RhAG mutation was obtained by venipuncture on the same day ektacytometry was done. All samples containing the whole cells from blood were washed with NaCl 0.9% and frozen in 20% glycerol solution as previously described [39]. These samples were stored by the Centre National de Référence des Groupes Sanguins (CNRGS). To remove glycerol, the blood cells were first washed in glycerol 10%, then in sorbitol 8%, and finally three times in NaCl 0.9%. Finally, RBCs derived from the last pellet were resuspended in ID-CellStab (Biorad, Marnes la Coquette, France) and washed in DPBS (Dulbecco’s Phosphate Buffer Saline Gibco, ThermoFisher Scientific, Illkirch, France) before being used in some of the experiments described below.

### 4.2. RBC Deformability Index and Elongation Index Measurements by Ektacytometry

One hundred μL of blood from each patient was run on an ektacytometer (LoRRca MaxSis, RR Mechatronics^®^, Zwaag, The Netherlands). Each patient was compared to a matched healthy control. Fresh whole blood cells suspended in a viscous aqueous polyvinylpyrrolidone solution were exposed to a fixed shear stress of 30 Pa, changing the RBC shapes from circular to elliptical, as previously described [60,61]. The deformation of RBC was then monitored as a continuous function of suspending medium osmolality. In particular, the deformability index (DI) and the elongation index (EI) of cells were determined along an osmotic gradient between 100 and 550 mOsm at a constant temperature of 37 °C.

Distinct features of OHSt RBC ektacytometry curve patterns were compared to that of controls, in particular: O_min_, reflecting the surface area-to-volume ratio and corresponding to the osmolality at which 50% of the RBCs hemolyzed during the regular osmotic resistance test; EI_max_, reflecting the membrane surface and corresponding to the maximal deformability index (DI) or elongation index (EI); and O′ or hyper points reflecting the hydration state of the cells and corresponding to the osmolality at which RBCs reach 50% of the DI_max_ in the hyper-osmolar area.

### 4.3. Cell Culture and Transfection

K562 (human erythroleukemia) cell line (American Type Culture Collection, Rockville, MD, USA) was grown between 2 × 10^5^ and 1.5 × 10^6^ cells/mL in Iscove’s Modified Dulbecco’s Medium (IMDM) glutamax (ThermoFisher Scientific, Illkirch, France) supplemented with FBS 10% and Gibco™ Antibiotic-Antimycotic at 37 °C with humid 5% CO_2_ atmosphere. Cells were counted by trypan blue counting in a Malassez cell or CASY cell counter (OMNI life science, Bremen, Germany) and used for less than 20 passages.

During the exponential growth phase, 10^6^ K562 cells were co-transfected by electroporation using the Amaxa^®^ Cell Line Nucleofector^®^ kit V (Lonza, Basel, Switzerland) with 1 µg human Stomatin Homology-Directed DNA Repair (HDR) plasmids containing a puromycin resistance gene and 3 µg of human Stomatin CRISPR/Cas9 KO plasmids and 3 µg of human Stomatin CRISPR/Cas9 KO plasmids (Santa Cruz Biotechnology, Dallas, TX, USA). Immediately after the transfection, cells were transferred into 2 mL of culture medium to which 5 µg/mL of puromycin (Santa Cruz Biotechnology, Dallas, TX, USA) were added 24 h post-transfection. The expression of both plasmids was stabilized for 3 weeks by keeping puromycin in the culture medium. Puromycin-resistant cells were then subjected to a limiting dilution cloning in 96-well microplates before expanding for flow cytometry analysis of several clones.

### 4.4. Stomatin and PANX1 Membrane Expression Analysis by Flow Cytometry

RBCs, diluted in DPBS to 1% hematocrit, were fixed in ice for 20 min in 1% paraformaldehyde (Sigma-Aldrich, St Quentin Fallavier, France)-0.025% glutaraldehyde (Sigma-Aldrich, St Quentin Fallavier, France), washed twice in DPBS and permeabilized in 0.1% n-Octyl-β-D-glucopyranoside (Bachem, Bubendorf, Switzerland) for 10 min at room temperature before incubation with primary antibodies.

K562 cells, diluted in DPBS at 2 × 10^6^ cells/mL, were fixed in ice for 20 min in 4% paraformaldehyde, washed twice in DPBS by centrifugation (1000× *g*, 5 min), and permeabilized in 0.1% saponin for 15 min at room temperature before incubation with primary antibodies.

All cell types were saturated in DPBS-BSA 1% for 1 h at 37 °C. Proteins were stained with 1/200 mouse anti-Stomatin E5 monoclonal antibody (Santa Cruz Biotechnology, Dallas, TX, USA) or 1/100 rabbit anti-PANX1 polyclonal antibody (Alomone Labs, Jerusalem, Israel) in DPBS-BSA 0.2% for 1 h at 37 °C. Both antibodies recognize intracellular epitopes.

After 2 successive washes in DPBS by centrifugation (1000× *g*, 5 min), secondary antibodies were coupled for 30 min at room temperature: for RBCs, 1/50 Allophycocyanine (APC) anti-rabbit (Jackson Laboratory, Bar Harbor, ME, USA), 1/100 Fluorescein Isothiocyanate (FITC) anti-mouse (Jackson Laboratory, Bar Harbor, ME, USA), and for cells, 1/50 APC anti-mouse (Jackson Laboratory, Bar Harbor, ME, USA) and 1/100 FITC anti-rabbit (Jackson Laboratory, Bar Harbor, ME, USA) in DPBS-BSA 0.2%. After 2 washes with DPBS, cells were analyzed using a flow cytometer (FACS Canto II, BD Biosciences, San Jose, CA, USA). Results were analyzed using FlowJo software (FlowJo, Ashland, OR, USA).

### 4.5. Stomatin and PANX1 Membrane Expression Analysis by Western Blot

Ghosts from washed RBCs were obtained by hypotonic lysis for 20 min at 4 °C in 5 mM sodium phosphate buffer pH8. After successive washes by centrifugation (27,000× *g* for 15 min at 4 °C) and removal of the supernatants that contain hemoglobin, the ghost pellets were collected. Protein quantification of these ghosts was obtained using Bicinchoninic acid assay by mixing samples with reagents of the Pierce™ BCA Protein Assay Kit (ThermoFisher Scientific, Illkirch, France) for 30 min at 37 °C and measuring absorbance at 562 nm using a plate reader (BioRad, Marnes la Coquette, France).

Ghost samples containing 20 µg of proteins were denaturized in 5X Laemli buffer (5% SDS, 5 mM Tris pH 6.8) with 2 mM β-mercaptoethanol (Sigma-Aldrich, St Quentin Fallavier, France) and were loaded in a 4–12% gradient acrylamide/bis-acrylamide gel (BioRad, Marnes la Coquette, France).

Proteins were transferred from acrylamide gel to nitrocellulose membrane by Trans-Blot Turbo system as preconized by the supplier (BioRad, Marnes la Coquette, France). The membrane was saturated in TBS (Tris Buffered Saline)-milk 5% for 1 h and incubated overnight at 4 °C with primary antibodies, anti-stomatin E5 (Santa Cruz Biotechnology, Dallas, TX, USA), and anti-PANX1 (Alomone Labs, Jerusalem, Israel) 1/1000 in TBS-milk 5%. Washes were carried out in TBS-Tween 0.1 %. HRP-conjugated secondary antibodies (Abliance, Compiègne, France) were incubated for 30 min at room temperature. After final washes in TBS, ECL Prime reagent (GE Healthcare Life Sciences, Velizy-Villacoublay, France) was added. The signal was detected by a Chemidoc MP apparatus (Biorad, Marnes la Coquette, France).

### 4.6. Proximity Ligation Assay

RBCs, previously fixed and permeabilized as described above, were washed twice by centrifugation at 1000× *g* for 5 min in DPBS and saturated for 1 h in Duolink^®^ Blocking Solution (Sigma-Aldrich, St Quentin Fallavier, France) and incubated with anti-stomatin or/and anti-PANX1 or neither for 1 h at 37 °C.

The proximity ligation assay was made according to the Duolink^®^ flowPLA Mouse PLUS /Rabbit MINUS kit-FarRed protocol (Duolink), using Duolink^®^ PLA Probes (1/5 MINUS anti-rabbit for anti-PANX1 and 1/5 PLUS anti-mouse for anti-Stomatin) and the FarRed Duolink^®^ Fluorescent Detection Reagent.

After 2 washes in DPBS, PLA positive (PLA+) RBCs were determined by detecting FarRed spots using Amnis Imagestream Mk II (Luminex, Minneapolis, MN, USA) and IDEAS software (EMD Millipore, Seattle, WA, USA) for the analysis.

### 4.7. Dye Uptakes

RBCs were washed twice by centrifugation (450× *g*, 5 min) in basal Krebs solution (in mM, 126 NaCl, 25 NaHCO_3_, 2.5 KCl, 1.2 NaH_2_PO_4_, 1.2 MgCl_2_, 2.5 CaCl_2_, pH 7.4, 300 ± 5 mOsm). In the RBC suspension diluted at 0.1% hematocrit, PANX1 was stimulated by substituting basal Krebs with Krebs in which Na+ was replaced by K^+^ in the presence of 2 mM of 5(6)-carboxyfluorescein (Sigma-Aldrich, St Quentin Fallavier, France) for 10 min. For inhibitions, 20 µM of carbenoxolone (Sigma-Aldrich, St Quentin Fallavier, France) or 100 µM of probenecid (Sigma-Aldrich, St Quentin Fallavier, France), or 0.1 µM of mefloquine (Bioblocks, San Diego, CA, USA) were added to the RBC suspension incubated for 20 min in the basal Krebs and then for 10 min with 2 mM of 5(6)-carboxyfluorescein in the K^+^ Krebs also containing the inhibitors. After 3 washes, carboxyfluorescein fluorescence in control and OHSt RBCs were analysed using a flow cytometer (FACS Canto II, BD Biosciences, San Jose, CA, USA).

In K562 transfected clones diluted at 1 × 10^6^ cells/mL in Tyrode Buffer (in mM, 135 NaCl, 5 KCl, 10 HEPES, 5 D-glucose, 1 MgCl_2_, 1.5 CaCl_2_, pH 7.4, 300 ± 5 mOsm), PANX1 channel, previously blocked or not, was then activated by adding 1/3 volume of water corresponding to a decrease of 80 mOsm. The inhibition was performed by adding 100 µM of carbenoxolone (or 500 µM of probenecid or 0.5 µM of mefloquine) 20 min beforehand. Non-viable cells were stained with Sytox blue (ThermoFisher Scientific, Illkirch, France), and apoptotic cells were stained with Lactadherin FITC (Haematologic Technologies, Essex Junction VT, USA) to exclude them from the analysis of dye uptake. After 5 min staining in 1/5000 TO-PRO-3 (ThermoFisher Scientific, Illkirch, France), the intensity of TO-PRO-3 fluorescence was analysed for intact cells (Sytox−/Lactadherin−) using a flow cytometer (FACS Canto II, BD Biosciences, San Jose, CA, USA). Three different measurements were performed for each clone and averaged before comparison.

In parallel, after the same hypoosmotic stimulation, caspase activation was evaluated by adding 1 µM of NucView 405 Caspase-3 substrate (Biotium, Fremont, CA, USA) and incubating at room temperature for 20 min.

### 4.8. ATP Release and Hemolysis

Immediately after fresh blood collection, plasma, platelets, and leukocytes were removed by centrifugation (900× *g* at 20 °C for 3 min). The supernatant and buffy coat were removed and discarded. Isolated red blood cells (RBCs) were resuspended and washed three times in an isoosmotic medium (in mM: 137 NaCl, 2.7 KCl, 2.5 Na_2_HPO_4_, 1.50 KH_2_PO_4_, 1.32 CaCl_2_, 1.91 MgSO_4_, 5 glucose, pH 7.4 at 25 °C, 300 mOsM) and supplemented with 0.1% BSA. Packed RBCs were resuspended in an isoosmotic medium to the corresponding final hematocrit.

Thawed RBCs were treated with Rejuvesol (Zimmer Biomet, Warsaw, IN, USA), diluted 6 times in DPBS with 5mM of Glucose (Sigma, St Quentin Fallavier, France) for 1 h at 37 °C, as previously described [44].

For ATP measurements, all reagents were of analytical grade. Bovine serum albumin (BSA), carbenoxolone, glucose, firefly luciferase (EC 1.13.12.7), and ATP were purchased from Sigma (St. Louis, MO, USA). D-luciferin was purchased from Invitrogen/ Molecular Probes Inc. (Eugene, OR, USA).

Total extracellular ATP (eATP) measurements were performed by online luminometry using the luciferin-luciferase reaction as described previously [16,23,24]. Briefly, 3 × 10^6^ RBCs were incubated in 50 μL of luciferin-luciferase reaction mix prepared in iso- or hypoosmotic (in mM: 113.6 NaCl, 2.7 KCl, 2.5 Na_2_HPO_4_, 1.50 KH_2_PO_4_, 1.32 CaCl_2_, 1.91 MgSO_4_, 5 glucose, pH 7.4 at 25 °C, 240 mOsm/kg) media. Experiments were performed with or without preincubation (20 min) of 100 μM CBX. Light emission was monitored for 20 min and then transformed into eATP concentration using a calibration curve. That is, at the end of each experiment, ATP from 1 to 32 μM was sequentially added to the assay medium from a stock solution of pure ATP dissolved in iso- or hypoosmotic medium. Results were expressed as eATP in pmol/10^6^ cells at 20 min post-stimulus.

Hemolysis was assessed in eATP measurement-paired samples by an enzymatic method described by Vazquez et al. [62] (Figure S2). Results were expressed as a percentage of hemolyzed RBCs (% Hemolysis). Hemoglobin concentration was used to estimate lytic eATP. The difference between total eATP and lytic eATP was calculated and denoted as non-lytic ATP.

### 4.9. PS Exposure and Caspase Activity on RBCs

To induce PS exposure and caspase activation, 1 µL of RBCs from 5 different donors was washed twice and resuspended at 0.1% hematocrit in RBC-2 buffer (in mM: 137 NaCl, 2.7 KCl, 2.5 Na_2_HPO_4_, 1.50 KH_2_PO_4_, 1.32 CaCl_2_, 1.91 MgSO_4_, 5 glucose, pH 7.4 at 25 °C, 300 mOsm) with Fenton solution (2 mM of H_2_O_2_ and 0.3 mM of FeSO_4_) during 1 h at RT. 1 µL of RBCs was resuspended in basal RBC-2 (300 mOsm/kg) or hypotonic RBC-2 (in mM: 113.6 NaCl, 2.7 KCl, 2.5 Na_2_HPO_4_, 1.50 KH_2_PO_4_, 1.32 CaCl_2_, 1.91 MgSO_4_, 5 glucose, pH 7.4 at 25 °C, 240 mOsm/kg) at 0.1% hematocrit. RBCs resuspended in the three different conditions (basal with Fenton, basal or hypotonic without Fenton) were stained with FLICA 660 caspase3/7 at a 1:60 ratio (*v/v*) during 20 min at 37 °C. After 2 washes in PBS-BSA 0.2%, RBCs were stained with Lactadherin-FITC 1/100 for 5 min at RT, and samples were analyzed by flow cytometry.

### 4.10. Statistics

Data were statistically analyzed using Prism Graph Pad 7.0 software (San Diego, CA, USA). Corresponding tests were indicated in the legend of each figure, as well as the number of independent experiments (N) used to estimate the mean and standard error of the mean. *p* values (>0.05: ns, ≤0.05: *, ≤0.01: **, ≤0.001: *** and ≤0.0001: ****) were obtained with a confidence interval of 95%.

## Data Availability

Experimental data of the results within this work would be available under request.

## Supplementary Materials

The following are available online at https://www.mdpi.com/article/10.3390/ijms23169401/s1. Reference [62] is cited in the supplementary materials.
