# Supplementary material for: Mechanosensitive Pannexin 1 Activity Is Modulated by Stomatin in Human Red Blood Cells
Source: Int J Mol Sci. 2022 Aug 20;23(16):9401. doi: 10.3390/ijms23169401 (PMC9409209; doi:10.3390/ijms23169401)
Supplement: Supplementary file 1 [file ijms-23-09401-s001.zip › ijms-1854406-supplementary.pdf]

Supplemental Material

Mechanosensitive PANX1 activity is modulated by stomatin in human Red Blood Cells

Sarah ROUGE[1], Sandrine GENETET[1], Florencia LEAL DENIS[2], Michael DUSSIOT[3], Pablo Julio SCHWARZBAUM[2], Mariano Anibal OSTUNI[1], Isabelle MOURO-CHANTELOUP[1]*

[1] Université Paris Cité and Université des Antilles, INSERM U1134, BIGR, F-75014 Paris, France
[2] Instituto de Química y Fisico-Química Biológicas "Prof. Alejandro C. Paladini", UBA, CONICET Facultad de Farmacia y Bioquímica, Junín 956, Buenos Aires, Argentina.
[3] Université Paris Cité, INSERM U1163, IMAGINE, F-75015 Paris, France
* Correspondence: isabelle.mouro-chanteloup@inserm.fr

Figure S1: PANX1 expression in control and OHSt RBC by flow cytometry

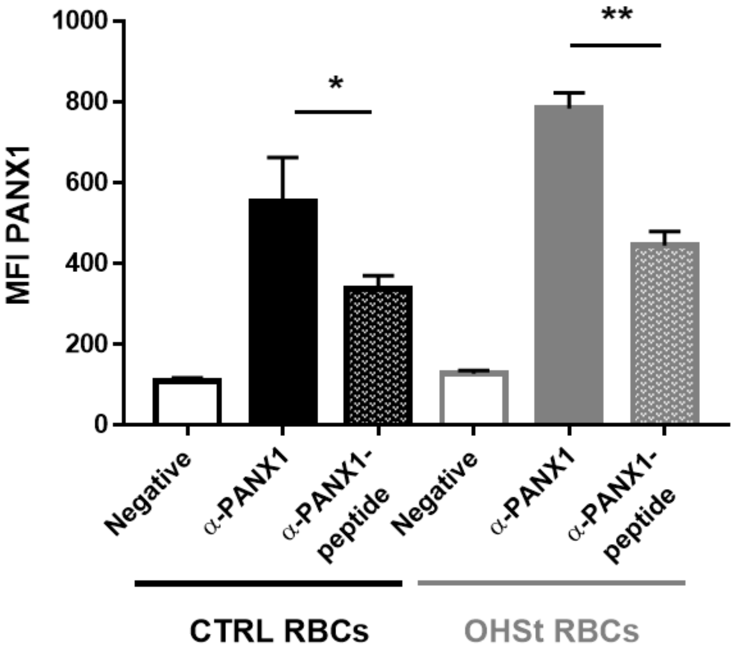

Prior to pannexin staining, 18-31 amino acid peptide of PANX1 (Alomone) were incubated 1h with α-PANX1 with a ratio of 1:10 (m/m). Two-way ANOVA test (paired for same population) * p values 0.0398, et ** p value 0.0079

Figure S2: Control and OHSt RBC hemolysis in different osmotic conditions

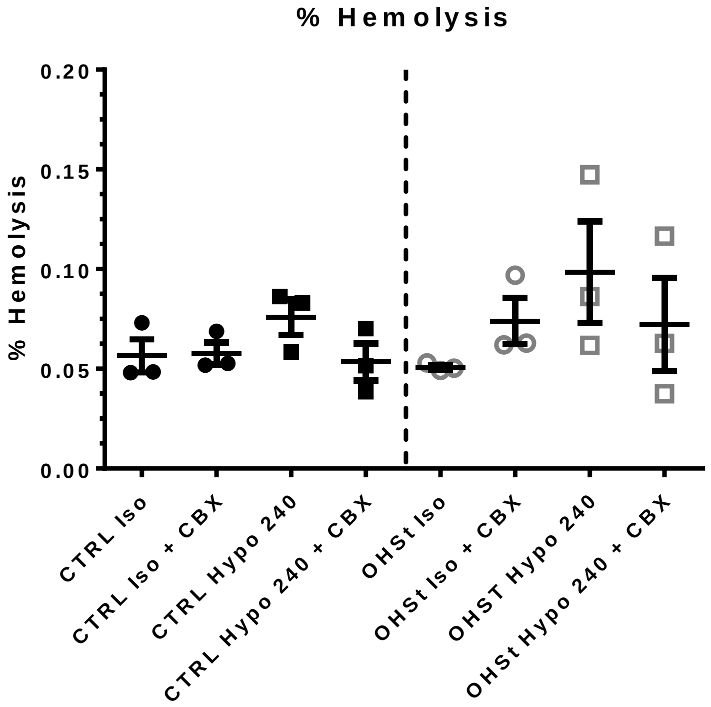

For each sample taken to assess eATP by luminometry, a parallel sample was taken to assess hemolysis. The RBCs were processed as described above except that the luciferin-luciferase has not been added. After 20 min incubation with or without CBX, samples were centrifuged at 12 600×g at 20 °C for 30 s and the presence of free hemoglobin in the supernatant was determined by an enzymatic method to detect microquantities of free hemoglobin as described by Vazquez et al. [1]. Results were expressed as percentage of total hemoglobin in the RBCs present in the sample.

Supplementary Table S1: Mean Corpuscular Volume (MCV), Mean Corpuscular Hemoglobin Concentration (MCHC) and Reticulocytes values of the RBCs from the three OHSt patients and controls (CTRL)

	Origin	MCV	MCHC (g/l)	Reticulocytes (G/l)
**CTRL**	CNRGS	80-100	330-360	20-120
**OHSt 1**	Nancy	132.5	273	269
**OHSt 2**	Grenoble	138	284	242.7
**OHSt 3**	Toulouse	144	247	ND

1. Vazquez, A.; Tudela, J.; Varon, R.; Garcia-Canovas, F., Determination of hemoglobin through its peroxidase activity on chlorpromazine. *J Biochem Biophys Methods* **1991,** 23, (1), 45-52.
